# Non-deletional alpha thalassaemia: a review

**DOI:** 10.1186/s13023-020-01429-1

**Published:** 2020-06-29

**Authors:** Ibrahim Kalle Kwaifa, Mei I. Lai, Sabariah Md Noor

**Affiliations:** 1 Haematology Unit, Department of Pathology, Faculty of Medicine and Health Sciences, University Putra Malaysia (UPM), Serdang, Selangor Malaysia; 2 Department of Haematology, School of Medical Laboratory Sciences, College of Health Sciences, Usmanu Danfodiyo University (UDU), Sokoto, North-Western Nigeria; 3 Genetics and Regenerative Medicine Research Centre, Faculty of Medicine and Health Sciences, Universiti Putra Malaysia(UPM), Serdang, Selangor Malaysia

**Keywords:** α-Thalassaemia, Molecular basis, Non-deletional mutations, Genotype-phenotype correlation

## Abstract

**Background:**

Defective synthesis of the α-globin chain due to mutations in the alpha-globin genes and/or its regulatory elements leads to alpha thalassaemia syndrome. Complete deletion of the 4 alpha-globin genes results in the most severe phenotype known as haemoglobin Bart’s, which leads to intrauterine death. The presence of one functional alpha gene is associated with haemoglobin H disease, characterised by non-transfusion-dependent thalassaemia phenotype, while silent and carrier traits are mostly asymptomatic.

**Main body:**

Clinical manifestations of non-deletional in alpha thalassaemia are varied and have more severe phenotype compared to deletional forms of alpha thalassaemia. Literature for the molecular mechanisms of common non-deletional alpha thalassaemia including therapeutic measures that are necessarily needed for the understanding of these disorders is still in demand. This manuscript would contribute to the better knowledge of how defective production of the α-globin chains due to mutations on the alpha-globin genes and/or the regulatory elements leads to alpha thalassaemia syndrome.

**Conclusion:**

Since many molecular markers are associated with the globin gene expression and switching over during the developmental stages, there is a need for increased awareness, new-born and prenatal screening program, especially for countries with high migration impact, and for improving the monitoring of patients with α-thalassaemia.

## Introduction

Thalassaemia is one of the most common genetic abnormalities, with an estimated carrier rate of 1–5% globally [1, 2]. It is a form of haemoglobinopathy characterised by mutations that resulted from either the absence or decreased expression of the affected globin gene. Approximately, 70,000 severely affected infants are born yearly [3]. Thalassaemia was initially confined to the tropical and subtropical regions, including the Mediterranean, Sub-Saharan Africa, Middle East, and Southern and Eastern Asia. However, regional migrations have increased the frequencies of thalassaemia in various parts of Europe and northern and southern parts of America. More than 90% of the thalassaemic individuals live in resource-poor and underdeveloped countries. These individuals usually die at an early age due to poor quality of life and lack of supportive healthcare [4]. This review aims to discuss more concisely on the molecular basis of α-globin gene expression with an emphasis on non-deletional mutation types of alpha thalassaemia. It also summarises the need for improved diagnosis and therapeutic measures, as well as awareness campaigns, coupled with genetic counselling, which was predicted to significantly improve the quality of life of the affected individuals.

### Globin gene expression and Haemoglobin production

**Fig. 1:**
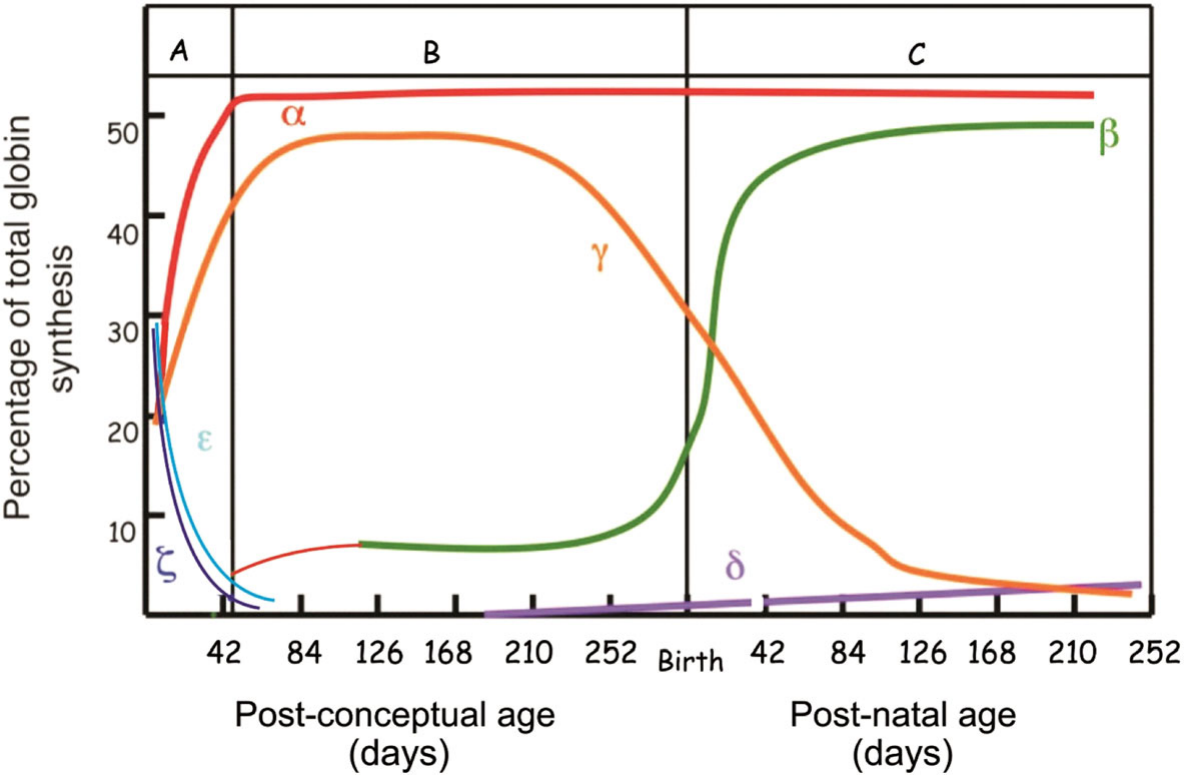
Haemoglobin synthesis during developmental stages. The age of the foetus/baby in days is represented in the x-axis while the y-axis indicates the percentage of the total globin genes expressed. The vertical lines indicate the time of birth. Within the first 42 gestational days, the embryonic genes and the first switching from ε- to γ-globin genes take place. The second switching of γ- to β-globin takes place soon after birth (Modified [4])

Haemoglobin (Hb) is a tetrameric molecule made up of 2 alpha-like (ζ or α) and 2 beta-like globin chains (ε, γ, δ or β), with each containing a heam group attached which serves as an oxygen carrier protein in the red cells [5]. There is approximately 250 million haemoglobins in one erythrocyte [6]. The alpha-globin gene cluster is situated at the short arm of chromosome 16 (16p13.3) (as 5′-ζ-μ-α2-α1–3′). Clusters of the alpha globin genes are arranged according to the order in which they are expressed during the developmental period. On the other hand, the beta-globin gene locus is located on chromosome 11 (11p15.5) as 5′-ε-Gγ-Aγ-δ-β-3′ [3]. Hb Gower-I (ζ_2_ε_2_), Hb Gower-II (α_2_ε_2_), and Hb Portland (ζ_2_γ_2_) are synthesised at early embryonic life, and foetal haemoglobin (HbF, α_2_γ_2_), which predominates throughout the foetal life. Postnatally, the foetal haemoglobin switches to HbA2 (HbA2, α_2_δ_2_) and HbA (α_2_β_2_) (⁓96–98%) (Fig. 1).

**Fig. 2:**
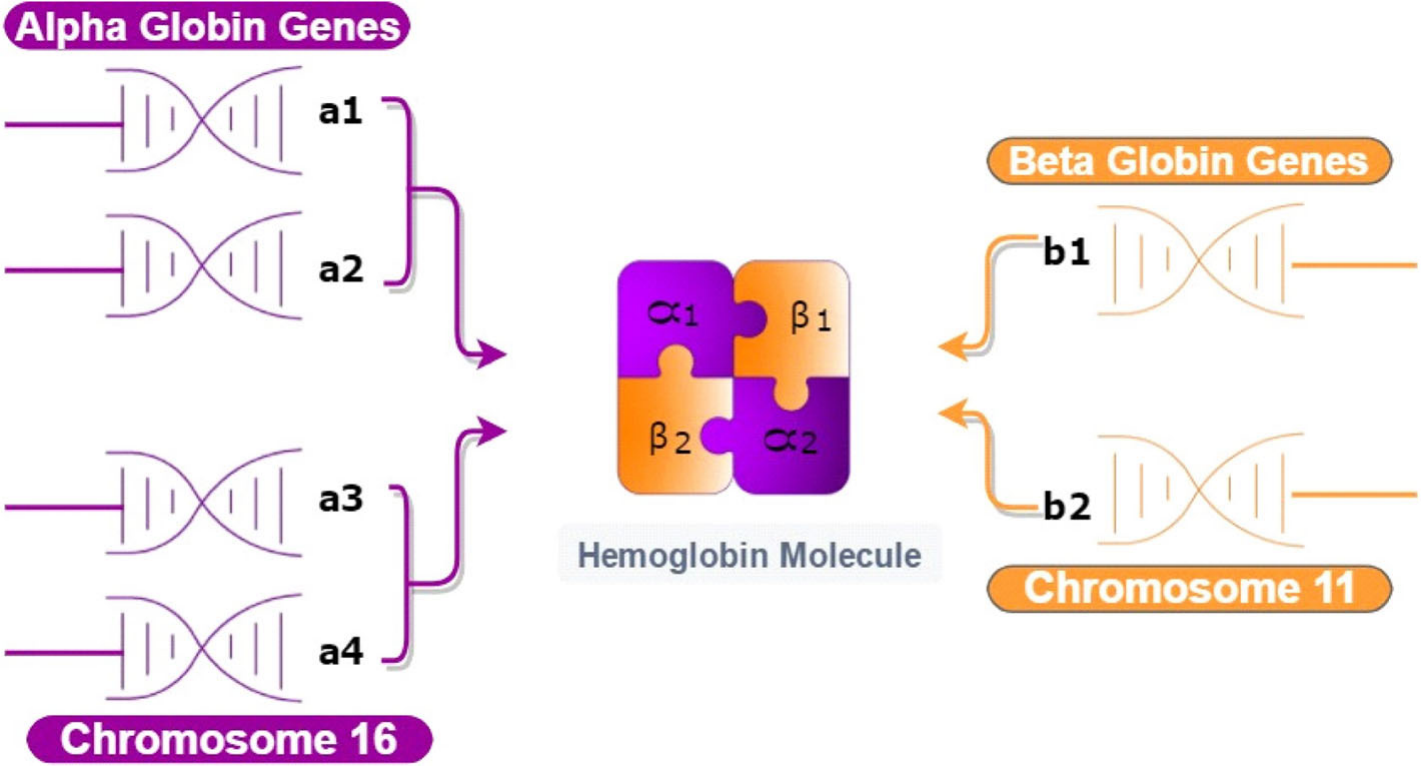
In normal haemoglobin synthesis, the alpha-globin genes produce only half of the products produced by the beta-globin genes to maintain the balance. An imbalance in the expression of globin genes from either side results in thalassaemia

Alpha-globin genes are expressed consistently at high levels from the beginning of foetal development. Hence, the effects of α-globin gene mutations are manifested throughout the foetal and adult life. This is quite different from the mutations of β-globin genes which exert their effects only approximately 6 month after birth. Generally, the collective production of alpha-globin chains from the four alpha-globin genes on chromosome 16 is estimated to be equalled to the total synthesis of the beta-globin chains derived from the two beta-globin genes on chromosome 11 (Fig. 2) [5].

### Mechanism of globin gene expression

Understanding the molecular basis of thalassemia requires comprehensive knowledge of the molecular mechanisms that coordinate and control the expression of the alpha and beta-globin genes. Alpha-globin expression is coordinated by four regulatory elements, known as enhancers, which are located at 10 to 48 kilobases upstream of the genes [3]. These enhancers are generally described as multispecies conserved sequences (MCS-R1–4), with underlying sites of DNase 1 hypersensitivity. Of these enhancers, MCS-R2 (HS-40) was reported to be the most effective regulatory enhancing element for the synthesis of alpha-globin [4]. Demethylation of repressive chromatin signatures coupled with promoter genes initiates the expression of the alpha-globin genes during erythroid differentiation. Transcription factors of the erythroid, including GATA-binding factor 1 (GATA1), nuclear factor erythroid 2 (NF-E2), stem cell leukaemia pentameric complex, and Kruppel-like factor1 (KLF1) are subsequently bound to the enhancers to promote alpha-globin gene expression [7]. Similarly, five enhancers of the β-globin genes, known as the locus control regions (LCR) have been described [3]. These are connected to active chromatin signatures (H3K4me3 and H3K27me3) and erythroid transcription factors, including LIM domain-binding protein1 (LDB1), GATA1, Friend of GATA1 (FOG1), KLF1, NF-E2, and stem cell leukaemia (SCL) factor on the β-globin promoter. Transcriptional repressors of γ-globin such as the B-cell lymphoma, also known as leukaemia 11A (BCL11A) protein were considered the most effective transcriptional repressors to direct the haemoglobin switching. Erythroid transcription factor KLF1 is also known to be involved with globin switching through direct activation of the beta-globin genes and promoting the synthesis of the γ-globin silencer BCL11A [8, 9]. Others include the haematopoietic transcription factor MYB which was reported to inhibit the γ-globin transcription in some ways by activating TR2/TR4 and KLF1 [3]. In recent times, the leukaemia/lymphoma-related factor (LRF) was recognised as a potent silencer to partially suppress the γ-globin genes [10, 11].

### Molecular basis α-Thalassaemia

More than 120 α-thalassaemia mutations have been reported and documented. Many of these mutations resulted from deletions at different lengths of the alpha-globin locus. Normally, an individual would have two pairs of the α-globin genes; two genes at each chromosome (αα/αα). But in alpha thalassaemia, a deletion may remove either one or both genes in a chromosome [3]. The two most common types of α^+^ thalassaemia (decrease in the expression of one or two of the alpha-globin genes) are –α^3.7^ and –α^4.2^. Defective synthesis of one or two α-globin genes results in mild to moderate changes in the red cell’s parameters. Alpha thalassaemia-α^0^ is mostly identified by the complete absence of α-globin genes and designated by the region where the condition was first discovered. For instance, −SEA, and –MED indicate that cases were first discovered in South East Asia and the Mediterranean, respectively [2]. Inactivation of three α-genes results in excessive β-globin chains production, forming β4 tetramers (HbH). These are extremely unstable variants which usually precipitate within the RBCs, resulting in HbH disease. HbH disease is characterised by variable degrees of haemolysis leading to haemolytic anaemia. The homozygous type of α^0^- thalassaemia (−−/−−) leads to the formation of γ-chains (γ4) tetramers, known as Hb Bart’s (γ4-Hb Bart’s); attributed to the hospital in the United Kingdom, the initial place where this condition was discovered. Hb Bart’s is characterised by a condition known as Hb Bart’s hydrops foetalis, which is associated with intrauterine death or death just after birth [2]. Occasionally, α-thalassaemia is also caused by deletions of the upstream enhancer elements (denoted as [αα]T). Even though normal α-globin genes are present, these deletions vary in length and have been reported to remove the critical enhancer MCS-R2 [12]. Additionally, very short deletions restricted to the MCS-R2 enhancer leaving all other enhancers and genes intact have also been identified to cause α-thalassemia [13] Common subtypes of alpha-globin genes disorders are summarised in Table 1.

**Table 1:** *Common alpha thalassaemia molecular disorders*

***Mutations***	***Genotype***	***Type and locations***
• Deletion at a single α-globin gene	-α or –α	-α^3.7^, −α^4.2^, (α)^20.5^ etc.
• Absence of α-globin genes on one chromosome	–	--^MED I^, −-^MED II^, −-^SEA^, −-^SPAN^, −-^FIL^, −-^THAI,^
• Mutations that affect the upstream regulatory elements	(αα)^T^	(αα)^RA^, (αα)^ALT^, (αα)^JX^
• Non-deletional mutations	α^T^α, αα^T^ or α^T^α^T^	α^CS^α, α^QS^α, α^ADANA^α, α ^IVS1(−5nt)^ α

MED I & II: Mediterranean I & II; SEA: South East Asia; FIL: Philippines; SPAN: Spain; THAI: Thailand; CS: Constant Spring; QS: Quong Sze; IVS1: Intervening Sequence; [αα]^T^: Deletion of the upstream enhancer elements; −α^3.7^: Single-gene deletion; −α^4.2^: Single-gene deletion; (α)^20.5^: Double-gene deletion; −-MED I: Double-gene deletion; −-MED II: Double-gene deletion; −-SPAN: Double-gene deletion; α2 IVS1: 5-bp deletion; ααα anti^-3.7^: Gene triplication; α1 cd 59: G > A (Hb Adana); −-FIL: Double-gene deletion; −-THAI: Double-gene deletion; −-SEA: Double-gene deletion; α2 cd 125: T > C (Hb Quong Sze); α2 cd 142: T > C (Hb Constant Spring).

### Genotypic and phenotypic correlation of α-thalassaemia

Most of the severity and haematological pictures observed in α-thalassaemia are associated with the extended range of cellular defects, described by partial or complete absence of the α-globin genes [14]. Deletion of one (−α/αα) or two α-globin (−−/αα, −α/−α) genes leads to asymptomatic forms of alpha thalassaemia known as silent carrier and thalassaemia trait, respectively. These are characterised by an imbalance between α and non-α globin chain syntheses. The genotype and phenotypic correlation are varying as summarized in Table 2. Patients have normal HbA_2_, mild to moderate microcytosis, and variable haemoglobin levels. HbH disease usually results from compound heterozygosity of α^+^ and α^0^ mutations (−−/−α) and is confined mostly within South East Asia, as well as the Mediterranean region. In contrast, atypical HbH disease state that results from non-deletional defects appears to be more severe compared to the deletional types. This could be attributed to the fact that non-deletional mutations are mostly associated with genomic regions that are critical for normal expression of α-globin genes; oligonucleotide insertions and deletion [14]. The clinical manifestations of Hb H disease look like those of α-thalassaemia intermedia, which is described by a considerable variable extent of anaemia. Hb H individuals may typically have hepatosplenomegaly, variable forms of jaundice, gallstones, and haemolytic crisis that may be fuelled by infections or drug therapy. While β-thalassaemia conditions are associated with ineffective erythropoiesis, in α-thalassaemia, the major cause of anaemia is due to haemolysis [14]. A complete absence of α-globin chain synthesis leads to Hb Bart’s hydrops foetalis, which is attributed to the inheritance of two α^0^ –thalassaemia phenotypes. The extent of anaemia, cardiovascular disorders, and other pronounced manifestations of this condition such as haemochromatosis usually result in intrauterine death (within 20–38 weeks of gestation) or shortly after delivery [15].

**Table 2:** *Alpha Thalassaemia Syndrome*

***Normal α-globin genes available***	***Significant phenotype***	***Genotype***	***Clinical findings***	***Potential risks to the foetus***
4	Normal	αα/αα	Clinically healthy	None
3	α-thalassaemia-silent traits	αα/−α –α/αα, α^T^α/αα, αα^T^/αα or αα/αα^T^	Normal Hb, low MCV but no clinical manifestation	HbH disease
2	α-thalassaemia carrier traits	−−/αα, −α/−α, -α^T^/αα, α^T^α/−α, α^T^α/α^T^α or α^T^α^T^/αα	Normally characterised by decreased Hb and MCV levels but no clinical manifestation	HbH disease, Hb Bart’s hydrops foetalis
1	HbH disease	-α/−−, αα^T^/−−, α^T^α/−−, α^T^α^T^/−α or αα^T^/−α^T^	Moderate to severe anaemia, low Hb and MCV	HbH disease, Hb Bart’s Hydrops Foetalis
0	Hb Bart’s hydrops foetalis	−−/−−, −α^T^/−− or α^T^α^T^/−−, −α^T^/−α^T^ or α^T^α^T^/−α^T^	Very severe anaemia and hypoxia characterised by intra-uterine death. Hydrops foetalis is associated with profound anaemia, low Hb, MCV, MCH, and jaundice. Others include high white blood cells count, increased bilirubin, normal platelet and reticulocyte counts, and hepatosplenomegaly.	Not compatible with life

α^T^: Non-deletional mutation.

### Molecular basis of non-Deletional type of alpha Thalassaemia

Over 70 forms of non-deletional mutations of α-thalassaemia have been identified and documented [16]. Non-deletional mutations include point mutations that affect genomic regions that are critical for the normal expression of α-globin genes. Point mutations that affect α_2_-globin gene appear to have more significant effects on the expression of the α-globin genes. Under normal circumstances, α_2_-globin gene expression is almost three times higher than α_1_-globin gene expression [14]. Non-deletional mutations can result in unstable haemoglobins that usually precipitate in the red cells, forming insoluble inclusion bodies and eventually damage or destroy the red cells membrane. Examples of non-deletional mutations of the HBA2 at termination codon include: Hb Constant Spring, α^IVS1(−5nt)^ α, the Hb Icara, Hb Koya Dora, α^TSaudi^α, poly-A α_2,_ Hb Quong Sze, Hb Seal Rock, Hb Bibba, Hb Chesapeake, Hb M-Boston, Hb Pakse′ as Hb Dartmouth, Hb Quong Sze, Hb Evora, Hb Heraklion, Hb Adana, Hb Aghia Sophia, Hb Petah Tikva, and Hb Suan Dok, among others. All these have their effects on the stop codon at position 142 of the coding sequence, leading to an extended α-globin chain and subsequently an extremely unstable Hb variant [17–19]. The molecular basis causing instability and decreased expression of α-globin genes may be associated with the cellular processing of an unstable mRNA, which has a reduced lifespan, or the unstable Hb variant may precipitate in the red cells resulting in haemolysis [16]. Since the unstable Hb variants may not have haem-haem interaction but they have high oxygen affinity that causes a decrease in the supply of oxygen to the tissues, the oxygen deprivation is thus characterised by haemolysis and anaemia. These would lead to dyserythropoietic marrow expansion, causing extramedullary erythropoiesis in the bone, liver, and spleen [2]. Summary of non-deletional mutations depending on stages involved in gene regulation of the affected gene and their distribution are as shown in Table 3.

**Table 3:** Summary of non-deletional mutations and their respective phenotypes. More concise lists of non-deletional mutations are presented in this link: (http://www.ithanet.eu/db/ithagenes; http://globin.bx.psu.edu/hbvar/menu.html) and are regularly updated [20–22]

Stages involved in gene regulation	Affected Sequence	Affected Gene	Specific Points of Mutation	Designated Name	Specific Region	Phenotype
**mRNA Processing**						
	IVS(a)	HBA2	IVSI(−5 nt)	Not Available	Mediterranean	α^+^
		HBA1	IVSI-1	,,,,	Thailand	α^+^
		HBA2	P1(AATAAG)	,,	Mediterranean	α^+^-α^0^
**Translation**						
		HBA2	InitATG>ACG	,,	Mediterranean	α^+^
		HBA2	InitATG>AG	,,	Vietnam	α^+^
		HBA2	InitATG>GTG	,,	Mediterranean	α^+^
		HBA2	InitATG>TG	,,	South East Asia	α^+^
	Exon II	HBA1	Cd51–55(−13 bp)	,,	Spain	α^+^
		HBA2	Cd90	,,	Middle East	α^+^
	Exon III	HBA1	Cd131	Hb Pak Num Po	Thailand	α^0^
	Termination	HBA2	Term Cd 427 T,143G	Hb Constant Spring	South East Asia	α^+^
		HBA2	Term Cd 428A, 143Ser	Hb Koya Dora	India	α^+^
		HBA2	Term Cd429A, 143Leu	Hb Paksé,	Laos and Thailand	α^+^
Protein Stability						
	Exon II	HBA2	Cd35	Hb Evora	Philippines and Portugal	α^+^-α^0^
		HBA1	Cd59	Hb Adana	China	α^+^-α^0^
	Exon III	HBA2	Cd125	Hb Quong Sze	China	α^+^

HBA1 and HBA2 are alpha-globin genes according to the HUGO nomenclature. *Cd* Codon, *P* Poly-A Signal, *term* Termination codon, *Del* Deletion, *int* Initiation codon, and *Hb* Haemoglobin. (Modified from Cornelis, L. Harteveld and Douglas, R. Higgs, 2010 [23]).

#### Haemoglobin constant spring (Hb CS)

Hb Constant Spring appears to be the most prevalent type of non-deletional mutations, resulting from the mutation of a ‘stop’ codon (α142, Term→Gln, TAA → CAA in α2). In this condition, a glutamine molecule is inserted. Thus, rather than the termination of the chain synthesis, it leads to the production of an α-globin chain with more amino acid residues [24, 25]. This results in an imbalance that favours the binding of the globin chains, which leads to an instability of the red cells. These red cells are often hydrated, which manifests with an abnormal MCV (Table 3) [16, 24, 26]. The concentration of Hb CS in the circulation is significantly reduced, with the estimation of 1–2% of the total Hb in the heterozygote state. Homozygous Hb CS might have similar phenotypes with thalassemia intermedia [26]. This condition was first discovered in Constant Spring, in Jamaica, from a Chinese family together with Hb H disease [25]. Hb CS was frequently reported in South East Asia, China, and the Mediterranean. It was recorded frequently in north-eastern Thailand (up to 10%) [26, 27] and 5–8% in Southern China [28]. Hb CS can also be seen in Malaysia, among the Malay, Chinese, and Indian populations at prevalence rates of 2.24, 0.66, and 0.16%, respectively. It was also identified among the aborigines (‘Orang Asli’ population) in West Malaysia [25]. In general, Hb CS appears to be the most dominant α-globin chain variant in South East Asia, followed by the relatively rare Hb QS in Malaysia and Singapore [29].

#### HbH-constant spring (HbH-CS)

Haemoglobin H-Constant Spring is a well-known identified non-deletional α-thalassaemia characterised by the combination of α^0^ and Hb CS **(−−/−α**^**CS**^**).** Generally, HbH-CS presents mild anaemia. However, very complicated haemolysis predisposing to acute haemolysis and severe foetal anaemia associated with hydropic features have been reported [30]. Also, an analysis of 145 paediatric patients with HbH Constant Spring has revealed that the clinical severity of this syndrome is widely variable. Many of these patients were classified as having a more severe phenotype since they had lower baseline Hb levels, needed frequent transfusions, and subsequently underwent splenectomy by 6 years of age. While other patients with the same genotype may have severe phenotypes and require intensive management. Several patients with HbH-CS did not require frequent transfusion or splenectomy and compensated quite well, having normal growth and pubertal development [16]. Detection of HbH-CS is very difficult by Hb electrophoresis due to decreased Hb H-CS level and the amount of unstable αCS mRNA in circulation [31]. An individual with Hb H-CS usually experiences frequent severe anaemia (Table 3), cholelithiasis, splenomegaly, and recurrent drop in haemoglobin because of intense sensitivity to oxidative stress. Clinical care for Hb H disease patients, especially the Hb H-CS should include regular blood transfusion, genetic counselling, enlightenment campaign on the associated complications, and prompt monitoring of the possible complications [32].

#### Haemoglobin Quong Sze (Hb QS)

Hb QS is a less common type of non-deletional mutation resulting from the HBA2-globin gene, in which amino acid eucine replaces proline (CTG → CCG, codon 125), leading to an extended α-globin chain. Patients of this condition also experience membrane dysfunction and mild to moderate haemolysis (Table 3) [26]. Hb QS (HBA2 c. 377 T > C) is any unstable haemoglobin variant recorded mostly in Southern China and Thailand [33]. It is one of the major alleles that causes non-deletional Hb H (β4) in the Chinese population [34].

#### Haemoglobin (Hb) Paksé

Hb Paksé is a rare form of non-deletional α-thalassaemia characterised by mutations at the termination codon of the HBA2-globin gene (TAA → TAT), resulting in an extended polypeptide. It is commonly found in central Thailand. Patients with this condition usually have low haemoglobin leading to mild to moderate anaemia, low MCV, MCH, and RBCs count (Table 3) [29].

#### Poly-adenylation (poly a) signal mutations

Poly A signal mutations are caused by variable base substitutions or deletion on the HBA2-globin gene. The incidence of these mutations prevails very highly in Greece, Saudi Arabia, and Turkey. These mutations are also attributed to the termination codon of the HBA2-globin gene, resulting in an extended polypeptide [29].

#### Haemoglobin (Hb) Koya Dora

Hb Koya Dora is found at a low frequency, which results from the mutations in the termination codon of the HBA2-globin gene, leading to an elongated polypeptide. Hb Kaya Dora was reported to be population-specific and about 10% incidence was recorded in the Koya Dora tribe of Andhra Pradesh in India [16]. This unstable Hb variant may form precipitates on the red cells membrane, causing haemolysis and ineffective erythropoiesis [16].

#### Haemoglobin (Hb) Chesapeake

Hb Chesapeake was first discovered in 1966, as a rare but with very high-oxygen-affinity [35]. It is an abnormal haemoglobin with a single α-chain substitution that has the molecular formula of α_2_92 Arg → Leuβ_2_A. The heterozygous forms are associated with polycythemia apparently to compensate for the increased oxygen affinity of this haemoglobin, which results in decreased liberation of oxygen in the tissues. This deletion affects the amino acid concerned with the α1-β2 chains contact and alters the rotational transition that occurs usually between the deoxygenated low-affinity state and the oxygenated high-affinity state. This prevents haemoglobin with the high-affinity relaxed state, causing hypoxia and compensatory erythropoiesis (Table 3). Patients with Hb Chesapeake have sporadic episodes of musculoskeletal (joint) pain but are expected to have a normal life. Significant frequencies of Hb Chesapeake were found in German and Irish families, as well as Japanese, French, and Afro-American [35].

#### Haemoglobin (Hb) ADANA

Haemoglobin Adana is a form of non-deletional alpha thalassaemia mutation, located at codon 59 of the HBA1 or HBA2-globin gene (GGC → GAC), leading to Gly → Asp replacement [36]. This substitution involves a glycine excess at a point of the E helix that is closely attached to a glycine residue of the B helix. This replacement significantly alters the stability and integrity of the cell’s molecule, leading to abnormal precipitates on the red cell membrane, which causes haemolysis and ineffective erythropoiesis [19]. This mutant variant was reported to be linked with a common α1-thalassaemia deletion [−(alpha) 20.5 kb] that results in a severe form of Hb H disease with anaemia. It is also characterised by decreased HbA2 level, elevated Zeta chain, increased Hb Bart’s, and a little of Hb H disease. The investigation for Hb Adana is very difficult, because the carriers may have normal haematological parameters [37]. Hb Adana can be detected in the laboratory by alpha-globin gene sequencing or PCR-based amplification refractory mutation system testing [38]. When Hb Adana combines with other α-globin deletions, this may yield various forms of phenotypes, ranging from mild anaemia (Table 3) (as in –α^3.7^ and –α^4.2^) [39, 40] to a more critical HbH-like condition (− − ^20.5^ and –α^4.2-QT (Q-Thailand)^ [41]. Hb Adana HBA2 point mutations that are co-inherited with a single α_1_ non-deletional mutation generally have severe Hb H-like manifestations (such as α_2_^CS^ [Constant Spring], α_2_^Paksé^, α_2_^IVS-II-142^, α_2_^IVS-II^, α_2_codon24, α_2_codon22, and rSNP 149,709 T > C) [41]. The incidence of Hb Adana is found to be low in countries such as Turkey (0.5–0.6%) [39], Iran and Iraq (1–2.5%) [42, 43], and China (1%) [43], while countries like Saudi Arabia (11.6%) [44], and Indonesia (16%) have a higher prevalence [40]. A variable incidence of 1–21.4% was recorded in various reports in Malaysia [45].

#### Haemoglobin (Hb) G Philadelphia

Hb G-Philadelphia is a stable and normal functioning oxygen carrier caused by a mutation at codon 16 of HBA1-globin gene (AAG → GAG), due to substitution of lysine by glutamic acid. It is characterised by a lower isoelectric point as compared to normal haemoglobin (HbA) and migrates faster than HbA at alkaline pH. Hb G-Philadelphia is similar to HbH but migrates with HbA at acidic pH [46]. It was first discovered by Rucknagel et al. in 1955 from an Afro-America family [47]. In the United State of America (USA), Hb G-Philadelphia is the highest frequent α-chain variant and identified from persons of African descent, with the carrier rate approximated to be 1 in 5000. Subsequently, it was identified in different ethnic groups including Africans, Caucasians, and Asians. It is also present in Italians (from Northern Italy), Indians, Sardinians, and a few Chinese families [46]. The Italian/Mediterranean mutation occurs on a normal functioning α-globin gene and is benign even when present in the homozygous form. Its trait (1 mutated gene) is completely silent. Genetic studies suggested that in most cases, the ΑG-P locus is the only functional locus on the affected chromosome. However, when α2-thalassaemia (3.7 kb deletion) occurs *in trans* (−α^G^/−α), the quantity of Hb G-P is increased to approximately 45%; this individual may have a distinct microcytosis and hyperchromasia (α2-thalassaemia) [46]. These patients may also have distinct microcytosis and hyperchromasia (α2-thalassaemia homozygotes) (Table 3). Hb G-Philadelphia in combination with α1-thalassaemia, although extremely rare, results in Hb H disease (−α^G^/−−) with 100% Hb G-Philadelphia. The co-inheritance of Hb G-Philadelphia (−α^G^/αα) with Hb S and /or Hb C is rather common. Patients characterised by Hb G-Philadelphia can have a complete four α-globin genes (α^G^α/αα) and 20–25% Hb G-Philadelphia with no haematological abnormalities [46].

### The way forward

#### Genetic Counselling and pre-natal screening

**Fig. 3:**
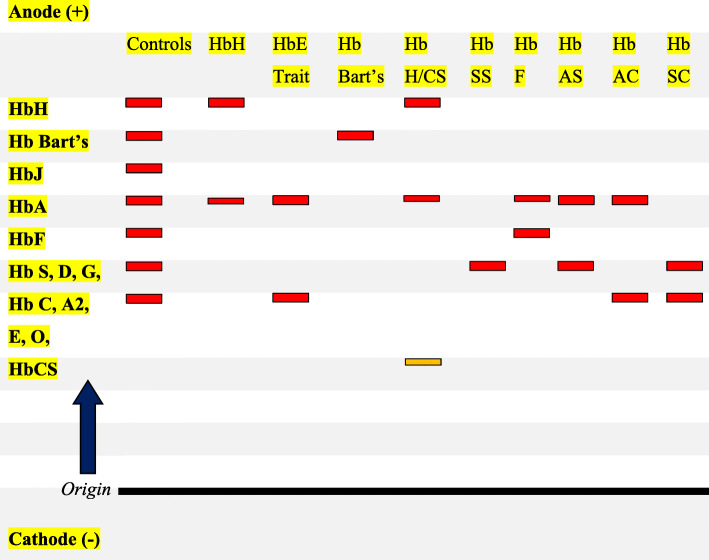
Diagrammatic representation of atypical haemoglobin cellulose acetate electrophoresis patterns at alkaline pH 8.4 to 8.6 showing some haemoglobin variants, including HbH and Hb Bart’s as fast-moving alpha thalassaemia bands. Others include: HbAA (Adult normal haemoglobin band), HbSS (sickle cell anaemia bands), Hb F (foetal haemoglobin bands), Hb SC (Sickle cell disease bands) and carrier traits (Hb E, D, C, S, O, G,CS etc). Hb CS, particularly, can only be identified when cellulose gel electrophoresis with heavy application is used

Knowledge on the molecular basis that regulates the expression of the α-globin genes is vital for the accurate understanding and differentiation of α-thalassaemia. Nationwide screening programmes, pre- and post-marital screening, including infant screening have been well developed. Accurate investigations of thalassaemia require screening of the population and identification of families who are at risk. Examinations of complete blood count for haemoglobin level and red cell indices, peripheral blood film morphology, together with Hb electrophoresis (i.e. by cellulose acetate electrophoresis patterns at alkaline as shown in Fig. 3), and modern DNA analysis will help for easy differentiation [1, 2]. Simple strip assay procedure for α-thalassaemia testing has also been developed. This method is cost-effective for the detection of the most frequent mutations observed, especially in patients with complicated prolonged microcytic anaemia [17]. Because a milder form of α-thalassaemia can be misinterpreted with IDA, since their red cell indices seem to be similar, a combination of iron studies must be assessed properly to discriminate between these two conditions [18]. Patients carrier to α^+^-thalassaemia, particularly the –α3.7 allele, may have similar haematological parameters and on this account DNA analysis is essential for the accurate diagnosis. The interrelationship between deletional and non-deletional forms of α-thalassaemia, α-globin genes mismatches, and deletions of the β-globin gene (leading to β-thalassaemia, Hb S, Hb E, etc) could make significant impacts on haematological and clinical severity observed in most patients. Consequently, the relationship between haematological analysis, haemoglobin electrophoresis by HPLC and/or cellulose acetate, molecular analysis of the α and β-globin genes and gene-cluster by the direct sequence are recommended as good laboratory practices for the accurate investigations of thalassaemia [48, 49]. Generally, genetic counselling of the inherited haemoglobinopathies, including α-thalassaemia disorders is crucial, at least to minimise the occurrence of Hb Bart’s hydrops foetalis syndrome, which may result in neonatal death and serious health complications to the mother during pregnancy. Genetic counselling will only make sense if the clinical outcome accurately differentiates between α-globin genotype and other related genotypes with the same manifestations as seen in the various forms of α-thalassaemia. With the recent development in genetic counselling, the identification of many genetic modifiers of α-thalassaemia was made easier, the effects on various phenotypes they caused were recorded, then the relatively cheaper high-throughput DNA testing should become more accessible [1].

#### Treatment and management of alpha thalassaemia

Alpha thalassaemia (except carrier and some α-thalassaemia traits) is generally a condition characterised by improved morbidity and mortality at an advanced age. Children with homozygous α-thalassaemia should be transfused immediately after delivery or receive intrauterine transfusions [50]. The treatment and prevention of α-thalassaemia syndromes should consider the following strategies.

##### Blood transfusion and iron chelation

In medical practices, blood is transfused to provide functional erythrocytes, alleviate ineffective erythropoiesis, and effectively minimise the pathophysiological processes in thalassaemia [51]. Modern blood transfusion practice and iron therapies promote the survival of thalassemia patients. Currently, most patients who are transfusion-dependent thalassaemia (TDT) can achieve near-normal Hb of around 8.7–12.0 g/dL. However, blood transfusion practices are still attributed to several complications or adverse effects. The most common significant effect of blood transfusion is the secondary iron overload [52]. Naturally, humans do not have simple means for iron excretion. With continued transfusion, excess iron will accumulate in body cells and plasma to the extent that the endothelial system, especially within the liver, would be incapacitated to hold the extra iron. The accumulation of iron is associated with the generation of reactive oxygen species and nitrite radicals, causing injury to lipid, proteins, DNA, and other cellular organelles. Iron accumulation is also attributed to a high risk of generating thrombosis, cardia associated strokes, hypothyroidism, hypertension, hypogonadism, osteoporosis, and renal dysfunctions [50]. Currently, a lot of newer techniques are now available for the investigations and management of iron overload. Assessment of serum ferritin level is commonly used and is recommended and affordable at least in most developing countries. The introduction of magnetic resonance imaging (MRI) for the non-invasive assessment of liver iron concentration and the use of superconducting quantum interference device (SQUID) are available but the technologies may be accessible and affordable to some limited countries globally [52]. There are three most commonly used iron chelators presently accessible for α-thalassaemic patients; subcutaneous or intravenous injection of deferoxamine, oral, and most recent film-coated tablet forms of deferiprone [52]. Of these, parenteral deferoxamine takes a greater advantage in patients with compensated heart failure. Nevertheless, the findings for novel iron chelators with specific efficacy, effectiveness, and safety continue. Finally, medical treatment is essential and adherence to stipulated administrative dosage is necessary for the achievement of the desired targets; these correlate to both successful management and patients’ survival as indicated in various studies [53].

##### Stem-cell transplantation for alpha thalassemia

Haemopoietic stem cell (HSCs) transplantation may be the most reliable curative therapy for patients with thalassaemia. Although not accessible and affordable to many resource-poor countries, the approach is now established for the definitive correction of defective haemopoietic stem cells, especially when matched sibling donors are available [2]. A recent development for the prevention and control of graft-versus-host with induced graft tolerance has discovered the utilisation of unrelated donors and umbilical cord blood as alternatives for haemopoietic stem cell transplantation [2]. Unfortunately, HSCs therapy may only be available to those with access to advanced medicine [1].

##### The used of pharmaceutical agents to treat alpha Thalassaemia

The benefits associated with medical procedures for the treatment of α-thalassaemia are to raise the level of α-globin gene expression or reduce the synthesis of f γ-globin or β-globin, thus maintaining the ratio balance of α-like and β-like globin chains while reducing the severity of alpha thalassaemia. Ideally, pharmacological agents should decrease the expression of γ-like and β-like globin genes [54]. However, several challenges are still yet to be resolved in epigenetic modification targeting drugs. Many of these drugs are linked with various genes throughout the genome and hence might have the potential to promote tumourigenesis. For example, Polycomb repressive complex 2 (PRC2) methylates lysine 27 in histone H3, a modification attributed with epigenetic genes silencing. This complex has a significant role in mediating cellular differentiation and development [3]. In haemopoietic stem cells, PRC2 depresses genes involved in cell cycle, cell differentiation, apoptosis, and self-renewal, but when PRC2 mutates, HSCs might not differentiate into mature red cells leading to the premature death of the cells [55, 56]. Also, in the ongoing clinical trial of mitapivat, a clinical proof-of-concept has been established based on a preliminary analysis of the Phase 2 trial of mitapivat (AG-348) and hence mitapivat was recommended to patients with non-transfusion-dependent thalassemia. Mitapivat is an investigational, first-in-class, oral, small-molecule allosteric activator of wild-type, and a variety of mutated pyruvate kinase-R (PKR) enzymes. The data obtained demonstrated that activation of wild-type PKR has the potential clinical benefit in thalassemia. The safety and tolerability profile observed in adults with pyruvate kinase deficiency supported the continued investigation of mitapivat treatment across severe, lifelong haemolytic anaemias such as pyruvate kinase deficiency, thalassemia, and sickle cell disease [57].

##### Gene therapy

Gene therapy was first proposed on thalassaemia as a good target because the defective expression of the globin genes directly affects the haemopoietic system, more specifically to the erythroid series [1]. Advancement in gene therapy will minimise the difficulties in getting compatible donors and the immunological complication usually characterised by the allogenic stem-cells transplantation. However, a lot of challenges have been observed in generating appropriate procedures, as the viral vectors can only contain a small portion of DNA. Fortunately, the use of retroviruses as vectors was changed to safer Lentiviral vectors, which gave long-term medication of the target globin on pre-clinical trials and ameliorated anaemia in a mouse of thalassaemia model. Even with the Lentiviral vectors, reports indicated that the viral vectors could transform into pro-oncogenes, with the consequent effects on a leukaemic transformation [58]. Quite many of the gene therapeutic procedures for β-thalassaemia and sickle disease have also been reported and documented [59].

## Conclusion

Various studies have indicated that the carriers of the alpha thalassaemia are discovered at a polymorphic prevalence (> 1%) in many of the tropical and subtropical populations. In regions where the carriers are very frequent, Hb H disease and Hb Bart’s hydrops foetalis would likely be observed with other alpha thalassaemia complications. The knowledge on the molecular basis associated with globin gene expression would facilitate the search for more new drugs to control the expression of these genes. Despite the advancements in modern technologies for thorough medical and scientific investigations of thalassaemia, efforts to progress in its management and to some extent accurate drug therapy yielded no appreciable results. Unfortunately, the public health care problem has been neglected in many jurisdictions, with catastrophic consequences for the affected families, coupled with the significant associated health management costs. Furthermore, the success of these developments might not give adequate opportunity for most of the patients to have such medications, especially those from resource-poor regions where advanced medical procedures are still lacking. The researchers should thoroughly investigate the molecular mechanisms associated with the expression of globin genes that will help to increase the knowledge in the armoury of the clinicians in the management of patients with thalassaemia until an ultimate cure becomes a reality. Since many molecular markers are associated with globin gene expression and switching over during the developmental stages, there is a need for increased awareness, new-born and prenatal screening program, especially for countries with high migration impact, and for improving the monitoring of patients with α-thalassaemia.

## Data Availability

As a review paper, the information was collected from previous journals (in-text cited) and all the references cited are included in the list of references.

## Abbreviations

Hb: Haemoglobin
α: Alpha
β: Beta
δ: Delta
γ: Gamma
ε: Epsilon
Hb Bart’s: Haemoglobin Bart’s
Hb H: Haemoglobin H Disease
HbF: Foetal Haemoglobin
ζ_2_ε_2_: Haemoglobin Gowe-1
H_2_S: Hydrogen Sulfide
α_2_ε_2_: Haemoglobin Gower-II
ζ_2_γ_2_: Haemoglobin Portland
α_2_β_2_: Haemoglobin A
α_2_δ_2_: Haemoglobin A2
SO_2_: Superoxide
Ζ: Zeta
IV: Intervening
Init: Initiation
CS: Constant Spring
HSC: Haemopoietic stem cells
NO: Nitric oxide
ROS: Reactive Oxygen Species
TNF-α: Tumour Necrosis Factor α
MCS-R1: Multispecies conserved sequences Region-I
NF-E2: Nuclear factor erythroid 2
KLFI: Kruppel-like factor1
LCR: Locus control regions
LDB1: LIM domain-binding protein1
FOGI: Friend of GATA1
SCL: Stem cell leukaemia
LRF: Lymphoma-related factor
MCV: Mean cell volume
RBCs: Red blood cells
SEA: South East Asia
MED: Mediterranean
H_2_O_2_: Hydrogen Peroxide
Term: Termination
MCH: Mean Cell Haemoglobin
TDT: Transfusion-dependent thalassaemia
iPSC: Induced-pluripotent Stem Cells
HPLC: High-Performance Liquid Chromatography

## References

[1] Higgs DR, Engel JD, Stamatoyannopoulos G (2012). Thalassaemia. Lancet.

[2] Taher AT, Weatherall DJ, Cappellini MD (2018). Thalassaemia. Lancet.

[3] Mettananda S, Higgs DR (2018). Molecular basis and genetic modifiers of thalassemia. Hematol Oncol Clin North Am.

[4] Mettananda S, Gibbons RJ, Higgs DR (2015). Α-globin as a molecular target in the treatment of Β-thalassemia. Blood..

[5] John SW, David HK, Chui MD (2001). The α-globin gene: genetics and disorders. Clin Invest Med.

[6] Jane BR, Noel M, Lisa AU, Michael LC, Steven AW, Peter VM (2015). Campbell Biology.

[7] Vernimmen D (2018). Globins, from genes to physiology and diseases. Blood Cells Mol Dis.

[8] Bauer DE, Kamran SC, Orkin SH (2012). Reawakening fetal haemoglobin: prospects for new therapies for beta-globin disorders. Blood..

[9] Zhou D, Liu K, Sun CW (2010). KLF1 regulates BCL11A expression and gamma- to beta-globin gene switching. Nat Genet.

[10] Masuda T, Wang X, Maeda M (2016). Transcription factors LRF and BCL11A independently repress expression of fetal haemoglobin. Science..

[11] Smith EC, Orkin SH (2016). Haemoglobin genetics: recent contributions of GWAS and gene editing. Hum Mol Genet.

[12] Higgs DR, Wood WG (2008). Long-range regulation of alpha-globin gene expression during erythropoiesis. Curr Opin Hematol.

[13] Wu MY, He Y, Yan JM (2017). Novel selective deletion of the major alpha-globin regulatory element (MCS-R2) causing alpha-thalassaemia. Br J Haematol.

[14] Grosso M, Sessa R, Puzone S, Maria Rosaria Storino PI (2012). Molecular Basis of Thalassemia, Anemia, *InTechOpen*.

[15] Weatherall DJ, Clegg JB. The Thalassaemia Syndromes, Fourth Edition. 2001. ISBN:9780865426641 |Online ISBN:9780470696705| 10.1002/9780470696705. Copyright © 2001 Blackwell Science Ltd.

[16] Fucharoen S, Viprakasit V. Hb H disease: clinical course and disease modifiers. Hematology Am Soc Hematol Educ Program. 2009;2009(1):26–34. 10.1182/asheducation-2009.1.26. 10.1182/asheducation-2009.1.26 20008179

[17] Karakaş Z, Koç B, Temurhan S, Elgün T, Karaman S, Asker G (2015). Hipokromik Mikrositer Anemili Olgularda Alfa Talasemi Mutasyonlarının Değerlendirmesi: İstanbul Perspektifi. Turkish J Hematol.

[18] Farashi S, Harteveld CL (2018). Molecular basis of α-thalassemia. Blood Cells Mol Dis.

[19] Wajcman HJ, Traeger-Synodinos I, Papassotiriou PC, Giordano CL, Harteveld V, Baudin-Creuza JO (2008). Unstable and thalassemic alpha chain haemoglobin variants: a cause of Hb H disease and thalassemia intermedia. Hemoglobin.

[20] Old J, Angastiniotis M, Eleftheriou A, Galanello R, Harteveld CL, Petrou M, Traeger-Synodinos J (2016). Prevention of Thalassaemias and Other Haemoglobin Disorders.

[21] Giardine B, Borg J, Viennas E, Pavlidis C, Moradkhani K, Joly P (2014). Updates of the HbVar database of human haemoglobin variants and thalassemia mutations. Nucleic Acids Res.

[22] Giardine B, Borg J, Higgs DR, Peterson KR, Philipsen S, Maglott D (2011). Systematic documentation and analysis of human genetic variation in hemoglobinopathies using the micro attribution approach. Nat Genet.

[23] Cornelis LH, Douglas RH (2010). α-thalassaemia: Review. Orphanet J Rare Dis.

[24] Pharephan S, Sirivatanapa P, Makonkawkeyoon S, Tuntiwechapikul W, Makonkawkeyoon L (2016). Prevalence of α-thalassaemia genotypes in pregnant women in northern Thailand. Indian J Med Res.

[25] Wee YC, Tan KL, Chua KH, George E, Jama T (2009). Molecular characterisation of Haemoglobin Constant Spring and Haemoglobin Quong Sze with a combine-amplification refractory mutation system. Malays J Med Sci.

[26] Singer ST (2009). Variable clinical phenotypes of α-thalassemia syndromes. ScientificWorldJournal..

[27] Tritipsombut J, Sanchaisuriya K, Phollarp P, Bouakhasith D, Sanchaisuriya P, Fucharoen G (2012). Micromapping of thalassemia and hemoglobinopathies in different regions of Northeast Thailand and Vientiane, Laos People’s Democratic Republic. Haemoglobin.

[28] Singsanan S, Fucharoen G, Savongsy O, Sanchaisuriya K, Fucharoen S (2007). Molecular characterization and origins of Hb constant spring and Hb Pakse' in southeast Asian populations. Ann Hematol.

[29] Viprakasit V, Tanphaichitr VS, Chinchang W, Sangkla P, Weiss MJ, Higgs DR (2004). Evaluation of alpha haemoglobin stabilizing protein (AHSP) as a genetic modifier in patients with β thalassemia. Blood..

[30] Charoenkwan P, Sirichotiyakul S, Chanprapaph P, Tongprasert F, Taweephol R, Sae-Tung R, Sanguansermsri T (2006). Anemia and hydrops in a fetus with homozygous haemoglobin constant spring. J Pediatr Hematol Oncol.

[31] Li D, Liao C, Li J (2007). Misdiagnosis of Hb constant spring (alpha142, term-->Gln, TAA-->CAA in alpha2) in a Hb H (beta4) disease child. Haemoglobin..

[32] Vichinsky E (2012). Advances in the treatment of alpha-thalassemia. Blood Rev.

[33] Wee YC, Tan KL, Chua KH, George E, Tan JAMA (2009). Molecular characterisation of Haemoglobin constant spring and Haemoglobin Quong Sze with a combine-amplification refractory mutation system. Malays J Med Sci.

[34] Yong YJ, Lui YH, Dong-Zhi L (2014). Screening and Diagnosis of Hb Quong Sze [HBA2: c.377T > C (or HBA1)] in a Prenatal Control Program for Thalassaemia. Hemoglobin.

[35] Brigitte G, Jacques S, Catherine B, Weiller PJ, Lena-Russo D, Disdier P. A new case of haemoglobin Chesapeake [5]. Haematologica. 2001;86(1):105. http://www.haematologica.it/2001_01/0105.htm. 11146582

[36] Cürük MA. Hb H (beta4) disease in Cukurova, Southern Turkey. Haemoglobin. 2007;31(2):265–71. 10.1080/03630260701297279. 10.1080/03630260701297279 17486510

[37] Aksu T, Yarali N, Bayram C, Fettah A, Avci Z, Tunc B (2014). Homozygosity for HBA1: c.179G > A: Hb Adana in an infant. Hemoglobin.

[38] Singh SA, Sarangi S, Appiah-Kubi A, Hsu P, Smith WB, Gallagher PG, Chui DHK (2018). Hb Adana (HBA2 or HBA1: c.179G &gt; A) and alpha thalassemia: Genotype-phenotype correlation. Pediatr Blood Cancer.

[39] Bozdogan ST, Yuregir OO, Buyukkurt N, Aslan H, Ozdemir ZC, Gambin T (2015). Alpha-thalassemia mutations in Adana province, southern Turkey: genotype-phenotype correlation. Indian J Hematol Blood Transfus.

[40] Nainggolan IM, Harahap A, Ambarwati DD (2013). Interaction of Hb Adana (HBA2: c.179G*>*A) with deletional and nondeletional (+)- thalassemia mutations: diverse haematological and clinical features. Haemoglobin..

[41] Tan JA, Kho SL, Ngim CF, Chua KH, Goh AS, Yeoh SL, George E (2016). DNA studies are necessary for accurate patient diagnosis in compound heterozygosity for Hb Adana (HBA2:c.179 > A) with deletional or nondeletional alpha-thalassaemia. Sci Rep.

[42] Alibakhshi R, Mehrabi M, Omidniakan L, Shafieenia S (2015). The spectrum of *훼*-thalassemia mutations in Kermanshah Province, West Iran. Haemoglobin.

[43] Akbari MT, Hamid M (2012). Identification of *훼*-globin chain variants: a report from Iran. Arch Iran Med.

[44] Chen FE, Ooi C, Ha SY, Cheung BM, Todd D, Liang R, Chan TK, Chan V (2000). Genetic and clinical features of haemoglobin H disease in Chinese patients. N Engl J Med.

[45] Yatim NF, Rahim MA, Menon K (2014). Molecular characterization of *훼*- and *훽*-thalassaemia among Malay patients. Int J Mol Sci.

[46] Arya V, Kumar R, Yadav RS, Dabadghao P, Agarwal S (2009). Rare haemoglobin variant Hb I Philadelphia in north Indian family. Ann Hematol.

[47] Rucknagel DL, Page EB, Jasen WN (1955). Haemoglobin I: an inherited haemoglobin anomaly. Blood..

[48] Traeger-Synodinos J, Harteveld CL, Old JMM, Petrou M, Galanello R, Giordano P (2015). EMQN best practice guidelines for molecular and haematology methods for carrier identification and prenatal diagnosis of the haemoglobinopathies. Eur J Hum Genet.

[49] Traeger-Synodinos J, Harteveld CL (2014). Advances in technologies for screening and diagnosis of hemoglobinopathies. Biomark Med.

[50] Musallam KM, Cappellini MD, Daar S (2014). Serum ferritin level and morbidity risk in transfusion-independent patients with β-thalassemia intermedia: the ORIENT study. Haematologica..

[51] Rund D (2016). Thalassemia 2016: modern medicine battles an ancient disease. Am J Hematol.

[52] Cappellini MD, Cohen A, Porter J, Taher A, Viprakasit V (2014). Guidelines for the management of transfusion-dependent thalassaemia (TDT).

[53] Delea TE, Edelsberg J, Sofrygin O (2007). Consequences and costs of noncompliance with iron chelation therapy in patients with transfusion-dependent thalassemia: a literature review. Transfusion..

[54] Helin K, Dhanak D (2013). Chromatin proteins and modifications as drug targets. Nature.

[55] Xie SY, Ren ZR, Zhang JZ, Guo X, Bin WQX, Wang S (2007). Restoration of the balanced α/β-globin gene expression in β654-thalassemia mice using combined RNAi and antisense RNA approach. Hum Mol Genet.

[56] Xie H, Xu J, Hsu JH (2014). Polycomb repressive complex 2 regulates normal hematopoietic stem cell function in a developmental-stage-specific manner. Cell Stem Cell.

[57] Agios Pharmaceuticals, Inc. (NASDAQ: AGIO) (2019). Agios Establishes Proof-of-Concept for Mitapivat in Non-transfusion-dependent Thalassemia Based on Preliminary Phase 2 Results at ASH.

[58] Cavazzana-Calvo M, Payen E, Negre O (2010). Transfusion independence and HMGA2 activation after gene therapy of human β-thalassaemia. Nature.

[59] Negre O, Eggimann AV, Beuzard JA, Ribeil P, Bourget S. Borwornpinyo*, et al.* gene therapy of the beta-hemoglobinopathies by Lentiviral transfer of the beta (a(T87Q))-globin gene, hum. Gene Ther. 2016;27:148–65. (http://www.ithanet.eu/db/ithagenes; http://globin.bx.psu.edu/hbvar/menu.html). 10.1089/hum.2016.007 PMC4779296 26886832

